# Targeting REV-ERBα for therapeutic purposes: promises and challenges

**DOI:** 10.7150/thno.43834

**Published:** 2020-03-04

**Authors:** Shuai Wang, Feng Li, Yanke Lin, Baojian Wu

**Affiliations:** 1 College of Pharmacy, Jinan University, Guangzhou, 510632, China; 2 Integrated Chinese and Western Medicine Postdoctoral research station, Jinan University, Guangzhou, 510632, China; 3 Guangzhou Jinan Biomedicine Research and Development Center, Jinan University, Guangzhou, 510632, China; 4 International Cooperative Laboratory of Traditional Chinese Medicine Modernization and Innovative Drug Development of Chinese Ministry of Education (MOE), College of Pharmacy, Jinan University, Guangzhou, 510632, China

## Abstract

REV-ERBα (NR1D1) is a circadian clock component that functions as a transcriptional repressor. Due to its role in direct modulation of metabolic genes, REV-ERBα is regarded as an integrator of cell metabolism with circadian clock. Accordingly, REV-ERBα is first proposed as a drug target for treating sleep disorders and metabolic syndromes (e.g., dyslipidaemia, hyperglycaemia and obesity). Recent years of studies uncover a rather broad role of REV-ERBα in pathological conditions including local inflammatory diseases, heart failure and cancers. Moreover, REV-ERBα is involved in regulation of circadian drug metabolism that has implications in chronopharmacology. In the meantime, recent years have witnessed discovery of an array of new REV-ERBα ligands most of which have pharmacological activities *in vivo*. In this article, we review the regulatory role of REV-ERBα in various types of diseases and discuss the underlying mechanisms. We also describe the newly discovered ligands and the old ones together with their targeting potential. Despite well-established pharmacological effects of REV-ERBα ligands in animals (preclinical studies), no progress has been made regarding their translation to clinical trials. This implies certain challenges associated with drug development of REV-ERBα ligands. In particular, we discuss the potential challenges related to drug safety (or adverse effects) and bioavailability. For new drug development, it is advocated that REV-ERBα should be targeted to treat local diseases and a targeting drug should be locally distributed, avoiding the adverse effects on other tissues.

## Introduction

REV-ERBα [also known as NR1D1 (nuclear receptor subfamily 1 group D member 1)] is a nuclear receptor and a core component of the molecular clock system. REV-ERBα was discovered in 1989 and its name was derived from genomic location on the reverse DNA strand of *v-erbA* oncogene (also called “thyroid hormone receptor α”) found in the avian erythroblastosis virus 1,2. About five years later, REV-ERBβ (NR1D2), the other member of NR1D subfamily, was identified 3. Due to the lack of an activation function 2 (AF2, a motif for recognition of co-activators) in ligand binding domain, REV-ERBα/β cannot activate gene transcription 4. Instead, REV-ERBα/β function as transcriptional repressors, and inhibit gene transcription by recruiting co-repressors nuclear receptor co‑repressor 1 (NCOR1) and histone deacetylase 3 (HDAC3) 5. REV-ERBα may play a more important role in regulating circadian rhythms as compared to its paralog REV-ERBβ. REV-ERBα-deficient mice show disrupted circadian rhythms characterized by a shortened period. However, impact of REV-ERBβ ablation on circadian rhythms is negligible 6.

Due to its role in direct modulation of clock and metabolic genes, REV-ERBα is first proposed as a drug target for treating sleep disorders and metabolic syndromes (e.g., dyslipidaemia, hyperglycaemia and obesity) in 2012 7. Recent years of studies uncover a rather broad role of REV-ERBα in pathological conditions including local inflammatory diseases, heart failure and cancers. Moreover, REV-ERBα is involved in regulation of circadian drug metabolism that has implications in chronopharmacology. In the meantime, recent years have witnessed discovery of an array of new REV-ERBα ligands most of which have pharmacological activities* in vivo*. In this article, we review the regulatory role of REV-ERBα in various types of diseases and discuss the underlying mechanisms. We also describe the newly discovered ligands and the old ones together with their targeting potential. In addition, the potential challenges associated with drug development of REV-ERBα ligands are discussed.

### REV-ERBα in molecular clock system

**Figure 1:**
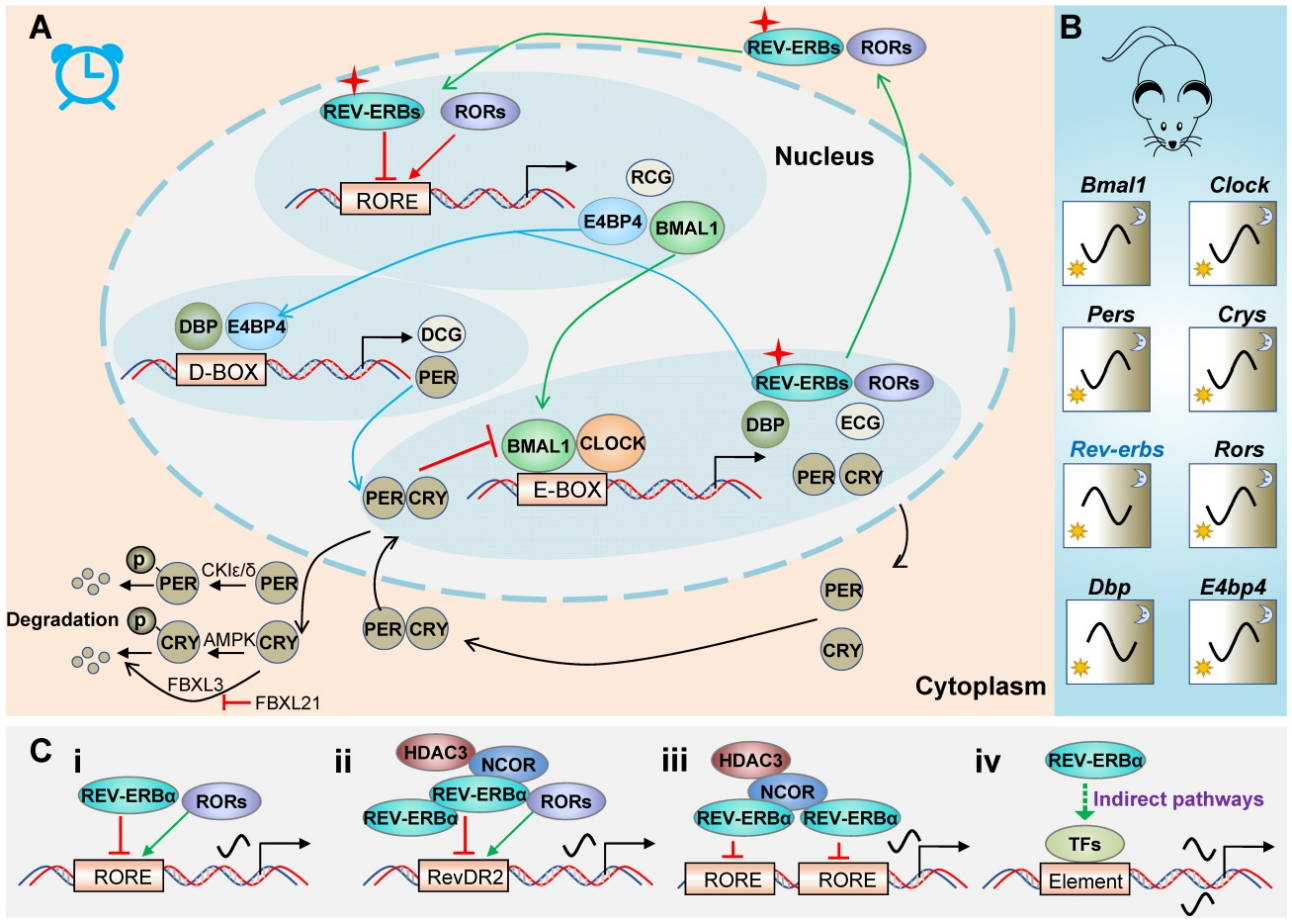
** REV‑ERBα in circadian clock system. (A)** Schematic diagram for molecular clock machinery. Mammalian molecular clock consists of three interlocked auto-regulatory feedback loops. The three loops are attained through PERs/CRYs (black lines), REV-ERBs/RORs (green lines) and DBP/E4BP4 (blue lines), respectively. **(B)** Circadian mRNA expression patterns of clock genes in mice. **(C)** General patterns for regulation of target genes by REV‑ERBα. REV‑ERBα directly regulates transcription of target genes via single RORE (i), RevDR2 (ii) or two adjacent ROREs (iii), and indirectly regulates gene transcription via other transcription factors (TFs) (iv).

REV-ERBα is a core component of circadian clock system in mammals. Mammalian molecular clock consists of three interlocked auto-regulatory feedback loops (Figure 1A) 8,9. The main loop is driven by BMAL1 (brain and muscle ARNT-like 1)/CLOCK (circadian locomotor output cycles kaput) heterodimer that induces the expression of E-box-controlled genes (ECGs) including *periods* (*PERs*) and *cryptochromes* (*CRYs*). Once reaching a high level, PER and CRY proteins move from the cytoplasm to the nucleus, and inhibit BMAL1/CLOCK activity. When the levels of PER and CRY proteins are reduced due to protein degradation, PER and CRY are dissociated from the BMAL1 /CLOCK complex and a new cycle of transcription is started. Degradation of PER and CRY proteins are controlled by casein kinases (CKIδ and CKIɛ) and adenosine monophosphate kinase (AMPK), respectively. These kinases tag the proteins via phosphorylation for ubiquitination and proteasome degradation 10-12. Alternatively, ubiquitination of CRYs can be mediated by FBXL3. However, FBXL21 forms an SCF E3 ligase complex to retain CRYs in the cytoplasm and protects CRYs from FBXL3-mediated degradation (Figure 1A) 13.

In the second loop (Figure 1A), BMAL1/CLOCK drives expression of REV-ERBs and RORs, which in turn respectively repress and activate *BMAL1* transcription and RORE/RevRE-controlled genes (RCGs) (Table 1). RCGs include genes involved in immune responses, metabolic homeostasis, cancers, nervous and cardiovascular systems. The third loop (Figure 1A) involves DBP and E4BP4 that regulate PER2 (an output gene from the main loop) and D-box controlled genes (DCGs). All clock genes are cyclically expressed although the patterns differ (Figure 1B). Of note, *REV-ERBα* (in mice) oscillates with a maximum level (zenith) at ZT6-10 and a minimum level (nadir) at ZT18-22 (Figure 1B). A large portion of clock controlled genes (CCGs, including *Bmal1* and *E4bp4*) are under the control of REV-ERBα (Table 1), and show characteristic patterns antiphase to *REV-ERBα* (Figure 1B).

REV-ERBα generally functions as a monomer and binds to a consensus half-site motif (A/G)GGTCA preceded by an A/T rich 5' sequence (named RORE or RevRE) on target gene promoters (Figure 1C) 3,14. In some cases, REV-ERBα can bind to direct repeats of RORE separated by 2 bp (RevDR2) as a dimer (Figure 1C). Moreover, two REV-ERBα molecules can separately bind to two adjacent ROREs and recruit co-repressors (i.e., NCOR1 and HDAC3) to regulate gene transcription (Figure 1C). Transcriptional repression mechanism of REV-ERBα may involve dynamic modulation of chromatin looping 15. REV-ERBα also acts to suppress gene expression at a distance by repressing the transcription of enhancer-derived RNAs (eRNAs) 16. In addition to direct regulation, REV-ERBα indirectly regulates gene transcription via repressing E4bp4 (Figure 1C) 17-19. This is supported by the fact that REV-ERBα and E4bp4 share a large number of target genes 20. REV-ERBα also indirectly regulates gene transcription by physically interacting with other transcription factors (e.g., HNF6, GR and NF-Y) (Figure 1C) 21-23.

### REV-ERBα and diseases

**Figure 2:**
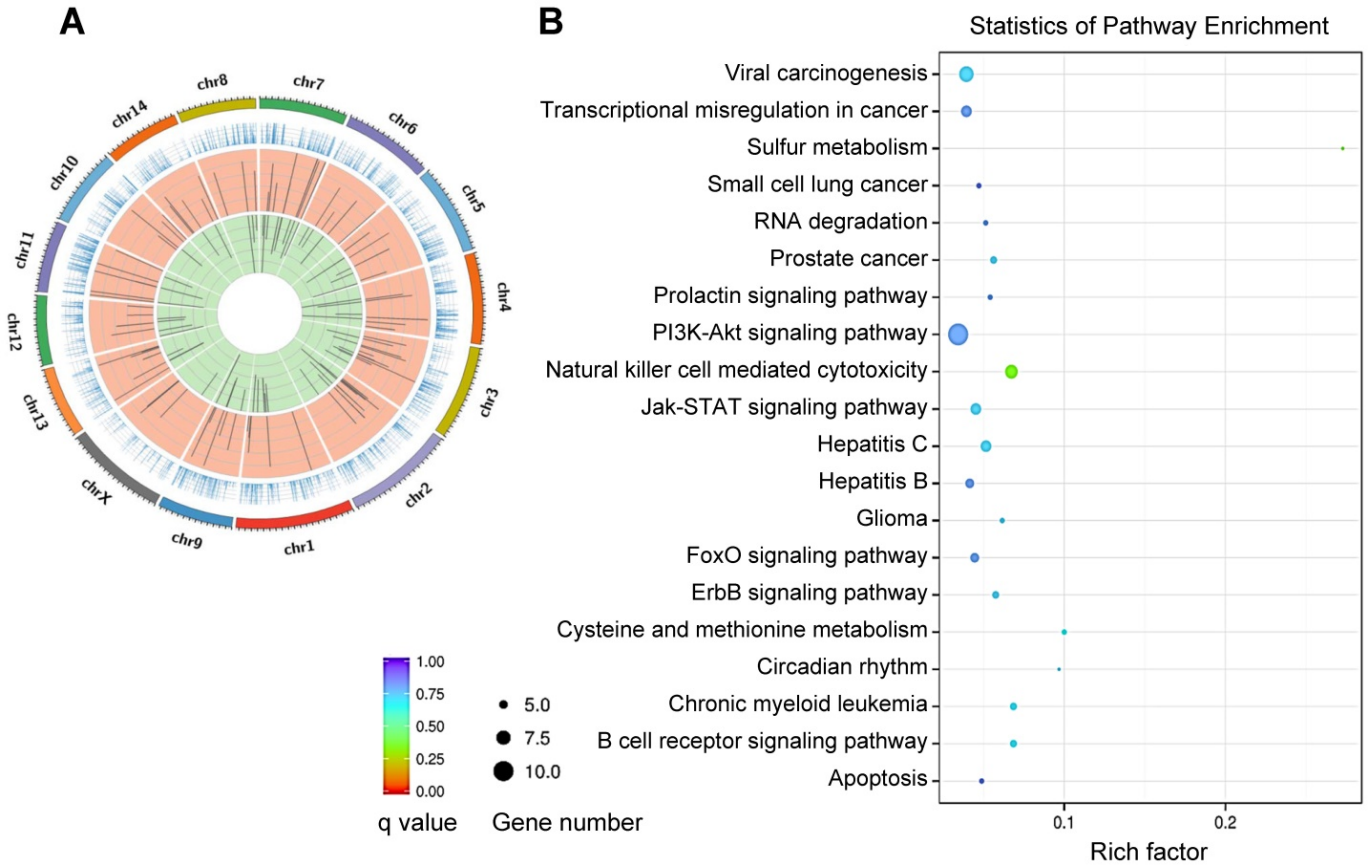
** REV‑ERBα controlled genes and pathways. (A)** Circos plot of differentially expressed genes between *Rev-erbα^-/-^*and wild-type mice, showing a disturbance in genome-wide gene expression. In the Circos plot, the outermost circle depicts the ideograms of each chromosome; the second circle represents gene expression levels; the third circle shows the distribution of the up-regulated genes; and the fourth circle shows the distribution of the down-regulated genes. **(B)** KEGG pathway analysis of Rev-erbα-induced differentially expressed genes in mouse liver (top 20 pathways are shown).

**Figure 3:**
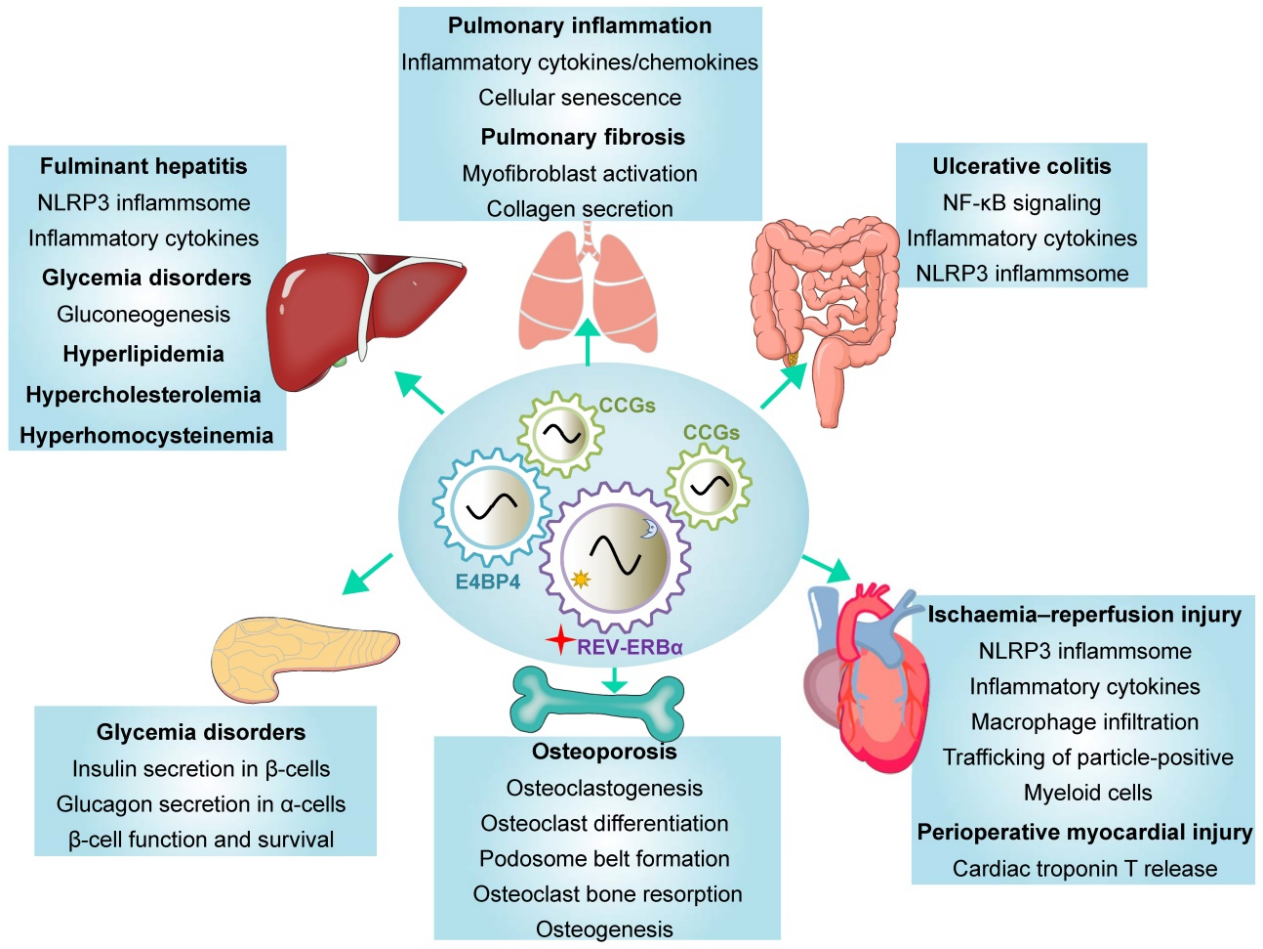
** Functions of REV‑ERBα in various tissues.** REV‑ERBα regulates clock-controlled genes (CCGs) to affect disease development in a tissue-specific manner. REV‑ERBα directly regulates target genes or indirectly regulates gene transcription via other transcription factors (e.g., E4BP4).

REV-ERBα has been implicated in regulation of a variety of diseases including inflammatory diseases, metabolic disorders and cancers (Table 2) 3,4. REV-ERBα expression is often times altered (expression changed and rhythm disrupted) during disease development 24,25. Reciprocally, dysregulation of REV-ERBα in humans and mice impacts the organism susceptibility to diseases 25-27. REV-ERBα knockout elicits disturbance in genome-wide gene expression (Figure 2A). The differentially expressed genes are associated with pathways involved in various pathological processes and diseases (Figure 2B). There is accumulating experimental evidence supporting REV-ERBα as a therapeutic target for diseases in the liver, lung, colon, pancreas, heart and bone (Figure 3 & Table 2).

### Role of REV-ERBα in inflammatory diseases

Inflammatory diseases often times exhibit time-varying severity or symptoms. For instance, patients with rheumatoid arthritis show diurnal variations in symptoms, as manifested by great joint pain, stiffness and functional disability in the morning 28,29. Patients received aortic valve replacement in the afternoon show alleviated perioperative myocardial injury compared to individuals received aortic valve replacement at other times of the day 30. Asthma is more severe in the early hours of the morning 31. Diurnal rhythmicity in the severity of inflammatory diseases may be associated with circadian REV-ERBα, a negative regulator of rhythmic inflammatory factors. Mice display dramatic daily differences in their susceptibility to LPS/D-GalN-induced fulminant hepatitis, with a lowest survival time at ZT16 that corresponds to a low REV-ERBα expression 27. Moreover, chronic colitis displays a diurnal rhythmicity in disease severity and its diurnal pattern is in an opposite phase to that of REV-ERBα 32. REV-ERBα ablation abrogates the diurnal rhythms of REV-ERBα-related inflammatory factors 25,32.

Accumulating evidence supports that targeting REV-ERBα is a promising approach for management of inflammations. REV-ERBα activation is shown to ameliorate ulcerative colitis 25,32,33, fulminant hepatitis 27, neuroinflammation 34,35, heart failure 36,37, myocardial infarction 38, experimental autoimmune encephalomyelitis 33,39, and pulmonary inflammation 29,40. Consistently, *Rev-erbα^-/-^* mice exhibit aggravated inflammations 25,27,33-40. Contrasting with a general anti-inflammatory role of REV-ERBα, Montaigne et al uncover a detrimental role of REV-ERBα in ischaemia-reperfusion injury, an inflammation-related disease 30. The authors show that REV-ERBα ablation or antagonism ameliorates ischaemia-reperfusion injury through promoting CDKN1a/p21 30. However, this study may not deny the anti-inflammatory effects of REV-ERBα because ischaemia-reperfusion injury is also determined by many other factors such as calcium overload, oxidative/nitrosative stress and endoplasmic reticulum stress in addition to inflammatory reactions 41.

**Figure 4:**
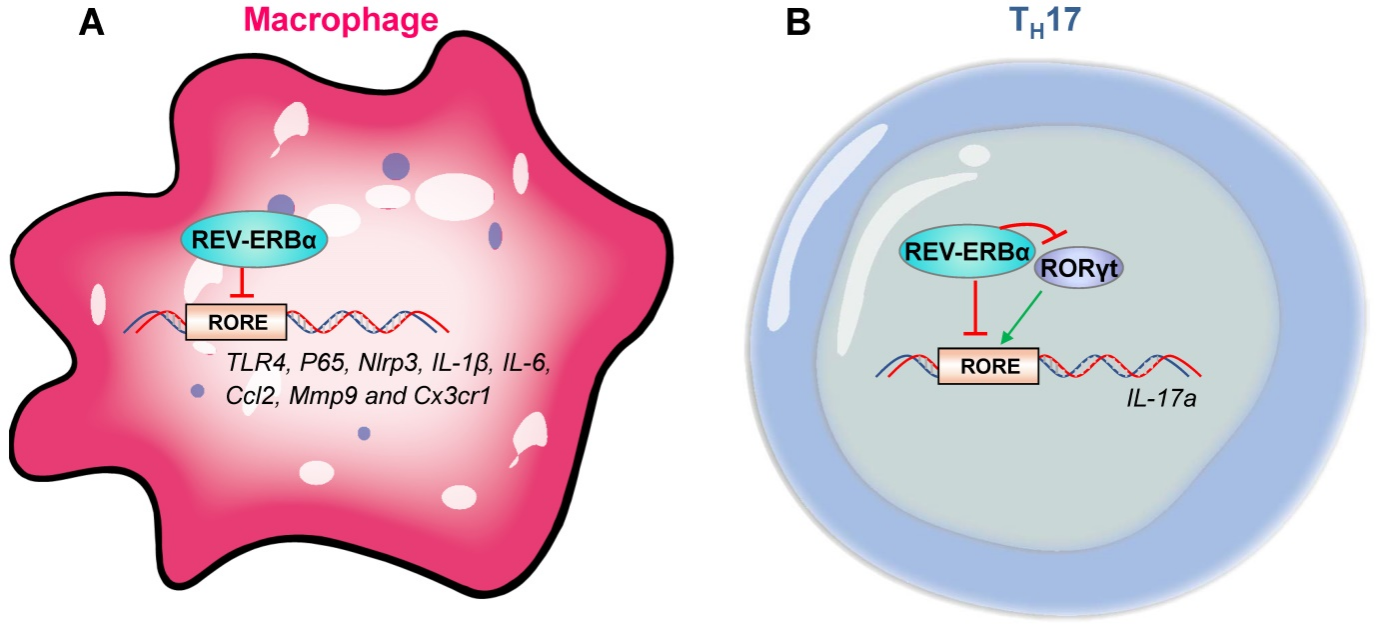
** REV‑ERBα regulates immune genes in macrophages and T_H_17 cells. (A)** In macrophages, a variety of immune genes (i.e., *P65*, *Nlrp3*, *IL-1β*, *TLR4*, *IL-6*, *Ccl2*, *Mmp9* and *Cx3cr1*) are controlled by REV-ERBα. **(B)** REV-ERBα negatively regulates T_H_17 cell development by competing with RORgt at the RORE of *Il-17a*. An additional mechanism involves REV-ERBα-mediated repression of RORgt.

The role of REV-ERBα in regulation of innate immune responses has been well established. REV-ERBα is involved in immune cell development, macrophage polarization, NF-κB signaling, transcription of inflammation-related genes (e.g., cytokine genes, chemokine genes and receptor genes) and activation of NLRP3 inflammasome. REV-ERBα impacts development of group 3 innate lymphoid cells (ILC3s) and secretion of related cytokines (i.e., IL-17 and IL-22) by controlling mitochondria 42. Activation of REV-ERBα impairs pro-inflammatory M1 phenotype and enhances anti-inflammatory M2 phenotype 43. REV-ERBα suppresses NF-κB signaling in human endometrial stroma cells and mouse macrophages/microglia cells, and down-regulates expressions of related genes, such as *Nlrp3*, *IL-6*, *IL-1β*,* IL-18*, *Tnfα* and *Ccl2* 25,34,35,44. In addition to an indirect regulation mechanism via NF-κB signaling, REV-ERBα directly regulates immune genes (e.g., *Nlrp3*, *IL-1β*, *TLR4*, *IL-6*, *Ccl2*, *Mmp9* and *Cx3cr1*) 16,25,27,45-48 (Figure 4A). Furthermore, REV-ERBα down-regulates Nlrp3 inflammasome activity to prevent ulcerative colitis, peritoneal inflammation, fulminant hepatitis and heart failure in mice 25,27,32,36.

The adaptive immune responses is also under the control of REV-ERBα. Similar to innate immune cells, T and B cells exhibit strong circadian oscillations in the blood, peaking in the rest phase 49. CD4+ and CD8+ T cells from murine lymph nodes exhibit a circadian rhythmicity in proliferation with a peak value in the evening 50. Pro-inflammatory CD4+ T helper 17 (T_H_17) are the adaptive correlates of ILC3s based on shared developmental requirement for the master transcription factor RORγt and secretion of IL-17 and IL-22 42,51. T_H_17 drives inflammatory responses in many autoimmune diseases, and is a well-established cell model for studying regulation of immunity by circadian clock 19,52,53. Effects of REV-ERBα on T_H_17 appear to be controversial. An early study reports that REV-ERBα drives T_H_17 cell differentiation and IL-17 production by repressing Nfil3 transcription 52. Supporting this, Farez et al report that melatonin inhibits ROR-γt and ROR-α expression in T_H_17 cells by regulating REV-ERBα-Nfil3 axis 54. However, later studies believe that REV-ERBα acts as a negative regulator of T_H_17 cell development by directly suppressing expression of IL-17 33,39. Chang et al proposed that the conflicting role of REV-ERBα in T_H_17 cells may be associated with its expression levels 39. When expressed at a low level, REV-ERBα promotes RORγt expression via suppression of the negative regulator Nfil3 52. At a high level, REV-ERBα competes with RORγt for binding to the promoter of *IL-17*, inhibiting gene transcription (Figure 4B) 39. Contrasting with an important role of REV-ERBα in T_H_17 cells, whether and how REV-ERBα regulates adaptive immunity in γδ T cells and regulatory T cells (with high REV-ERBα expressions) remain poorly explored 39.

Inflammation may lead to necrosis of parenchymal cells and promote the development of fibrosis. REV-ERBα agonist SR9009 alleviates CCl_4_-induced fibrosis in mice, as evidenced by reduced collagen deposition and decreased fibrotic gene expression 55. Consistently, REV-ERBα suppresses the development of pulmonary fibrosis in mice in a recent study 56. Lungs from *Rev-erbα^-/-^*mice reveal increased αSMA and collagen-1, two markers of myofibroblast activation. REV-ERBα agonist suppresses myofibroblast differentiation and collagen secretion in tissues from pulmonary fibrotic patients 56.

### Role of REV-ERBα in metabolic disorders

Many metabolic genes exhibit significant circadian oscillations. Chronic disruption of circadian rhythms (e.g., by shift-work and sleep deprivation) have detrimental effects on cell metabolism, resulting in metabolic disorders such as diabetes, hyperlipidemia and obesity. There is accumulating evidence supporting a critical role of REV-ERBα in regulation of cell metabolism and metabolic diseases.

### Glucose metabolism

REV-ERBα is implicated in glucose homeostasis and diabetes development due to its critical roles in regulation of glucose *de novo* synthesis and of pancreatic α/β-cell function. Activation of REV-ERBα reduces the levels of cellular and plasma glucose 7,57,58. Consistently, REV-ERBα-deficient mice show an increased level of plasma glucose 6,59. Yin et al demonstrate that REV-ERBα modulates glucose metabolism through regulating gluconeogenic rate-limiting enzymes phosphoenolpyruvate carboxykinase (PCK) and glucose‑6‑phosphatase (G6Pase) in human hepatoma cells and in primary mouse hepatocytes 57. Accordingly, REV-ERBα can be targeted to alleviate glycemia disorders and diabetes 59-61. In addition to the gluconeogenesis, REV-ERBα has a regulatory role in functions of pancreatic α and β-cells. At high glucose concentrations, REV-ERBα regulates glucose-induced insulin secretion in β-cells probably via modulation of the exocytotic process 62,63. At low glucose levels, REV-ERBα promotes glucagon secretion in pancreatic α-cells through AMPK/Nampt/Sirt1 pathway 63,64. Moreover, REV-ERBα enhances the survival and activity of β-cells under diabetogenic conditions 65.

Intracellular glucose levels oscillated in a circadian manner 66. REV-ERBα has been implicated in regulation of glucose rhythm. Up-regulation of REV-ERBα by MYC leads to reduced level of Bmal1 and loss of circadian glucose metabolism 66. CDK1-FBXW7 promotes REV-ERBα degradation in mouse liver, disrupting the circadian rhythmicity in glucose homeostasis 67. Dietary iron modulates heme synthesis and REV-ERBα activity, thereby altering the circadian rhythm of hepatic gluconeogenesis 68.

### Lipid metabolism

REV-ERBα-deficient mice exhibit a defect in lipid metabolism, causing increases in liver triglyceride and free fatty acids 6,69,70. Activation of REV-ERBα results in reduced triglyceride and free fatty acids in mice 7,71. The lipid-lowering effect is associated with transcriptional repression of ApoC-III (playing a key role in triglyceride metabolism by preventing catabolism of triglyceride-rich particles) and Elovl3 (elongation of fatty acids to produce very long-chain fatty acids) 69,72. Regulation of lipogenic genes by REV-ERBα may require tethering factors such as HNF6 73.

Cholesterol level mainly depends on the biosynthesis and elimination process. HMGCR (3-hydroxy-3-methylglutaryl-CoA reductase) and Cyp7A1 (cholesterol 7α-hydroxylase) are the rate limiting enzymes for cholesterol biosynthesis and catabolism, respectively. REV-ERBα was initially shown to regulate cholesterol catabolism (or biosynthesis of bile acids) and hypercholesteremia via a positive control of Cyp7a1 74,75. However, consensus was not reached regarding the mechanisms of action. REV-ERBα may regulate Cyp7a1 through E4bp4/Shp or Insig2/Srebp. By contrast, a recent study demonstrates that REV-ERBα inhibits Cyp7a1 expression via repressing Lrh-1 (an activator of Cyp7a1) that is supported by an early study 7,76. Additionally, the effects of REV-ERBα on cholesterologenesis may also involve modification of cholesterol biosynthesis-related genes such as *Hmgcr* 71.

The relationships between *REV-ERBα* polymorphisms and predisposition to obesity have been also recognized. The *REV-ERBα* rs2071427 polymorphism modulates body fat mass in both adult and adolescent people 26. Another polymorphism rs2314339 (in the intron of *REV-ERBα*) was associated with obesity in two cohorts from Mediterranean and North American population 77. Recently, *REV-ERBα* polymorphism rs939347 is shown to modulate body fat mass in men, suggesting a gender-specific role of REV-ERBα in the development of obesity 78.

### Amino acid metabolism

Homocysteine (a sulfur-containing amino acid) metabolism proceeds through two major pathways: remethylation to methionine and a two-step transsulfuration to cysteine 79. REV-ERBα plays a crucial role in homocysteine metabolism and ammonia clearance 80. Mechanistically, REV-ERBα regulates homocysteine catabolism through direct trans-repression of Bhmt, Cbs, and Cth, and ammonia clearance through inhibition of C/EBPα (CCAAT/enhancer-binding protein α) transactivation of Arg1, Cps1, and Otc 80. It was proposed that targeting REV-ERBα represents a new approach in management of homocysteine- and ammonia-related diseases 80.

### Bone metabolism

Disruption of circadian rhythms is associated with osteoporosis and abnormal bone metabolism, suggesting a close association between circadian clock and bone metabolism 81,82. REV-ERBα is periodically expressed in murine calvarial bones 83. Activation of REV-ERBα suppresses RANKL-induced podosome belt formation and inhibits osteoclast bone resorption, thereby ameliorating ovariotomy-induced bone loss 84. Further, REV-ERBα regulates osteoclastogenesis via inducing FABP4 84. In addition, REV-ERBα inhibits osteogenesis by repressing the expression of bone sialoprotein in bone mesenchymal stem cells 85. Therefore, REV-ERBα plays a pivotal role in maintaining metabolic homeostasis of bone by regulating osteoclastogenesis and osteogenesis.

### Role of REV-ERBα in cancers

REV‑ERBα has been implicated in development and progression of gastric cancer 86. It is associated with clinicopathological factors including poor differentiation, T stage, TMN stage and lymph node metastasis in human gastric cancer 86. Patients with low REV‑ERBα expression exhibit poor prognosis compared with patients with high REV‑ERBα expression, indicating REV‑ERBα as a prognosis factor for gastric cancer 86. REV‑ERBα activation induces apoptosis in human gastric cancer cells and in 3T3-L1 preadipocytes 86,87.

Anti-proliferative effects of REV-ERBα have been observed in human breast and gastric cancer cells 88,89. Activation of REV-ERBα suppresses proliferation of breast cancer cells regardless of ER or HER2 status. REV-ERBα appears to pause the cell cycle of the breast cancer cells prior to M phase through direct targeting of cyclin A2 88. By contrast, anti-proliferative effects of REV-ERBα are attained through inhibiting glycolytic flux and pentose phosphate pathway in another study 89. To be specific, REV-ERBα inhibits the expression of PFKFB3 and G6PD (two genes involved in glycolysis and pentose phosphate pathway), thereby interfering with glycolytic flux and pentose phosphate pathway 89.

Sulli et al proposed that pharmacological targeting of REV-ERBα is a promising strategy for cancer treatment 90. The anticancer activity of SR9009 and SR9011 (two REV-ERBα agonists) affects a number of oncogenic drivers (such as HRAS, BRAF and PIK3CA) and persists in the absence of p53 and under hypoxic conditions 90. Activation of REV-ERBα causes cancer cell death but does not affect the viability of nontransformed cells. Mechanistically, REV-ERBα suppresses de novo lipid biosynthesis through repression of two key rate-limiting enzymes (i.e., fatty acid synthase and stearoyl-CoA desaturase 1), resulting in a deficiency of oleic acid 90. Moreover, REV-ERBα activation inhibits autophagy as evidenced by accumulation of p62 and lysosomes and a reduction in autophagosomes 90. Additionally, SR9009 impairs viability of NRAS-driven naevi and glioblastoma growth and improves animal survival 90. Taken together, REV-ERBα regulates cancer development via suppressing proliferation, *de novo* lipogenesis and autophagy, and inducing apoptosis in cancer cells. REV-ERBβ is also shown to be overexpressed in breast cancer cells in the study of De Mei *et al* 91. Unlike REV-ERBα, REV-ERBβ displays a cytoprotective action 91. The cytoprotective function of REV-ERBβ appears to operate downstream of autophagy blockade 91. The authors demonstrated that dual inhibition of both REV-ERBβ and autophagy may be an effective strategy for eliciting cytotoxicity in cancer cells 91.

### Role of REV-ERBα in drug metabolism

Metabolism (biotransformation catalyzed by drug-metabolizing enzymes) is a main defense mechanism of the body against xenobiotic threats, and regarded as a key determinant of pharmacokinetics (and drug exposure) and therefore of pharmacological effects. On the other hand, toxic metabolites may be generated from metabolism reactions, causing adverse effects and disfavoring new drug development. Many drug-metabolizing enzymes (DMEs) are expressed in a circadian time-dependent manner 18. Circadian expressions of DMEs most likely result in dosing time-dependent pharmacokinetics and therefore in time-varying drug effects (toxicity and efficacy) 18. Indeed, over 300 medications showed time-varying effects (up to a 10-fold magnitude) 92,93. Oxaliplatin, a drug for treating colorectal cancer, is a well-documented case that was initially halted in phase I clinical trial due to safety problem (extensive toxicity) 94. However, the safety of oxaliplatin was latter established in phase I and phase II clinical trials by using the knowledge of chronopharmacology 95,96. Therefore, integrating chronopharmacology with drug development processes would help to reduce adverse effects and maximize efficacy via dosing time optimization 97.

Cyp7a1 is a REV-ERBα-controlled enzyme that catalyzes the first and rate-limiting step of bile acid biosynthesis (or cholesterol catabolism). REV-ERBα regulates expression of Cyp7a1 and its activity is a determinant of the efficiency of bile acid biosynthesis 74-76. The mechanism by which REV-ERBα regulates Cyp7a1 is controversial. Indirect mechanisms involving one or two transcriptional factors such as Shp, E4bp4, Srebp and Lrh-1 have been proposed by multiple groups of investigators 74-76. Moreover, REV-ERBα is a negative regulator of Ces2, a family of phase I enzymes that play an important role in xenobiotic clearance and lipid metabolism 98. E4bp4 regulates Ces2 enzymes through inhibition of the repressor activity of REV-ERBα, thereby impacting the metabolism and pharmacokinetics of the Ces2 substrate CPT-11 (or irinotecan, a first-line drug for treating colorectal cancer).

**Figure 5:**
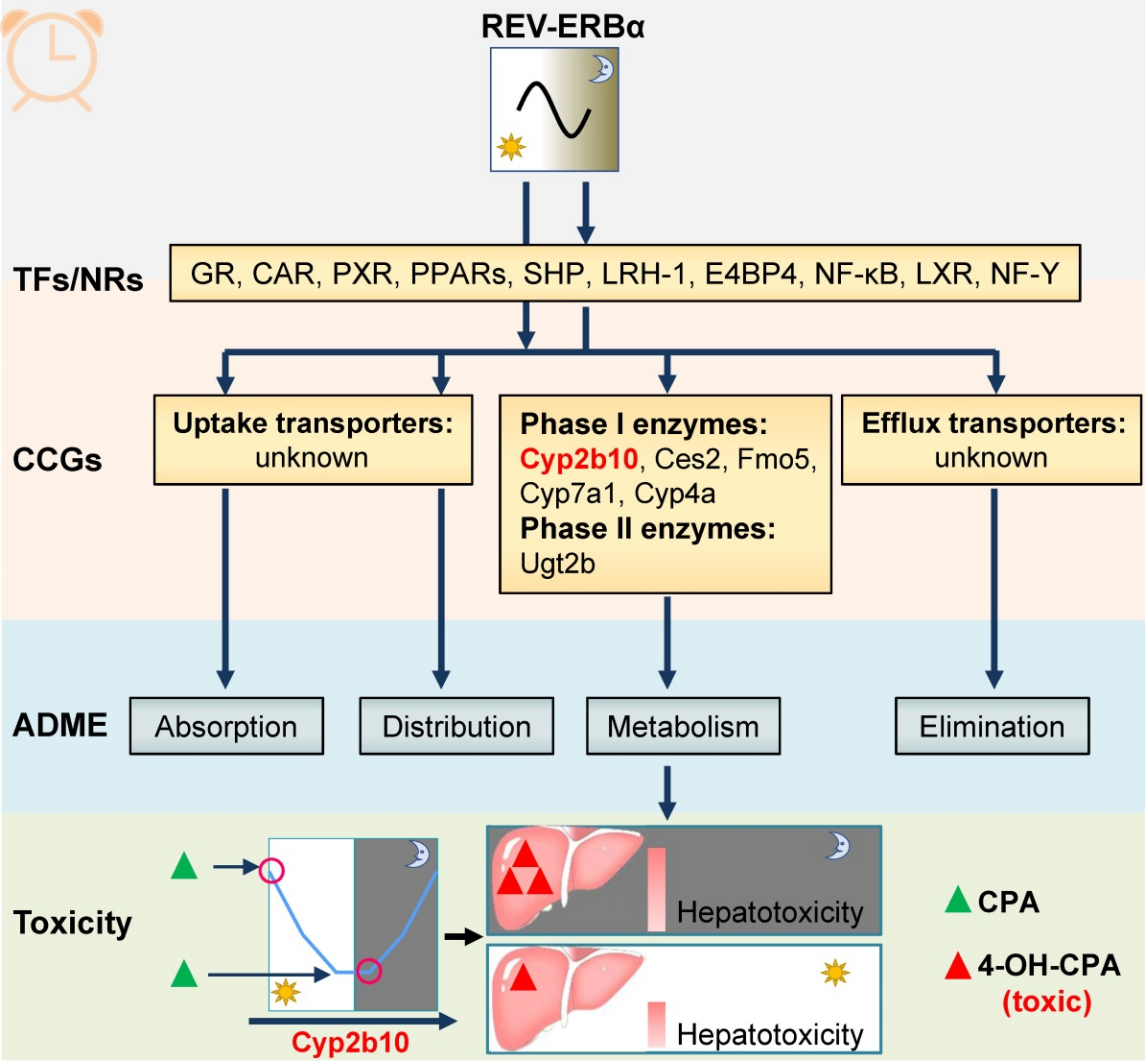
** REV‑ERBα is involved in chronopharmacokinetics and chronotoxicity via regulating drug-metabolizing enzymes (DMEs).** REV-ERBα directly or indirectly regulates clock-controlled genes (CCGs) involved in drug metabolism. The DMEs consists of “phase I enzymes” and “phase II enzymes”. Circadian expressions of these genes result in dosing time-dependent pharmacokinetics and therefore in time-varying drug effects (toxicity and efficacy). For instance, Cyp2b10 (a Rev-erbα target) metabolizes cyclophosphamide (CPA) to its toxic (4-hydroxylated) metabolite 4-OH-CPA. The CPA chronotoxicity is associated with time-varying generation of 4-OH-CPA caused by diurnal Cyp2b10 expression.

REV-ERBα transcriptionally regulates cycling enzymes such as Ugt2b, Cyp2b10 and Cyp4a10/14 (Figure 5) 99-101. Regulation by REV-ERBα contributes to circadian expressions of these enzymes and to circadian metabolism and pharmacokinetics of drug substrates such as morphine 99. Additionally, circadian enzymes and metabolism usually leads to chronotoxicity. For instance, Cyp2b10 metabolizes cyclophosphamide (CPA) to its toxic metabolite 4-OH CPA 101. CPA hepatotoxicity is dosing time-dependent in mice with high levels of toxicity at ZT2/22 and low levels at ZT10/14 101. The CPA chronotoxicity is associated with time-varying formation of 4-OH CPA caused by diurnal Cyp2b10 expression (Figure 5) 101.

### Overview of ligands for REV-ERBα

**Figure 6:**
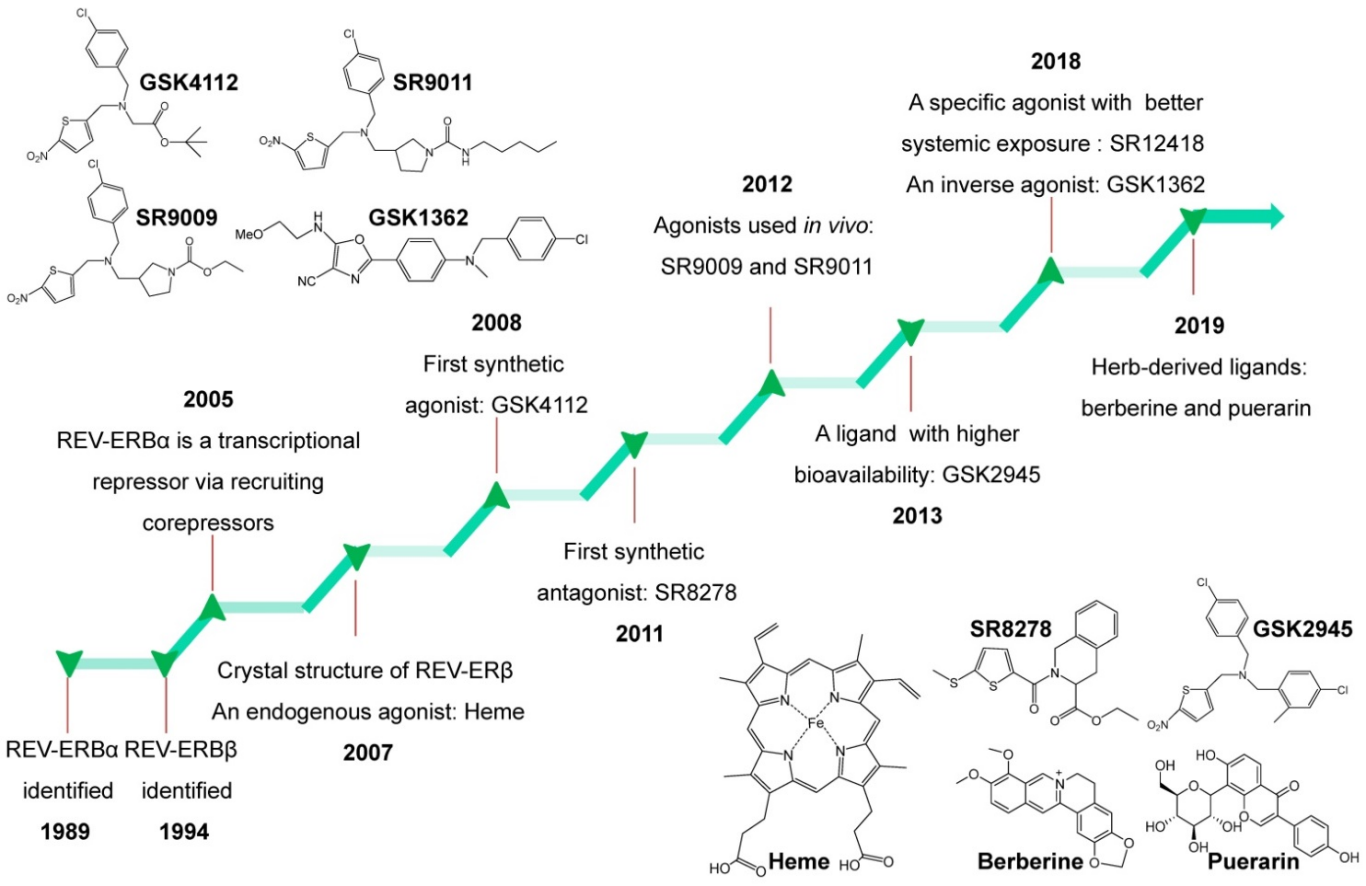
** Historical timeline for discovery of REV-ERBs and development of representative ligands from 1989 to 2019.** Chemical structures of all ligands except SR12418 (unavailable) are shown in the figure.

REV-ERBα is a nuclear receptor that can be targeted by small-molecule ligands. Burris and co-workers performed an excellent review of REV-ERB ligands in 2014 4. Recent years have witnessed discovery of an array of new REV-ERBα ligands most of which have pharmacological activities* in vivo* (Figure 6). In the following sections, we describe the newly discovered ligands and the old ones together with their targeting potential (Figure 6). It is noteworthy that all these REV-ERBα ligands most likely also act on its paralog REV-ERBβ.

### Heme

Heme was identified as an endogenous ligand (agonist) for REV-ERBs In 2007 102,103. Heme binds to ligand-binding pocket of REV-ERBs via interactions with two residues, a histidine on helix 11 and a cysteine on helix 3 104. Additionally, bulky hydrophobic residues in the ligand-binding pocket form hydrophobic interactions with the porphyrin ring of heme molecule 4. As a prototypical agonist, heme has been used to verify the effects of REV-ERB activation on gene expressions in *in vitro* studies 105. Manipulation of heme homeostasis is shown to alter circadian gene expression and glucose metabolism, highlighting the role of heme and REV-ERBs in circadian biology 68,106.

### GSK4112

Discovery of heme as a REV-ERB ligand opens the door for the development of more potent and effective synthetic ligands. GSK4112 (also known as SR6472) is the first synthetic ligand for REV-ERBs, identified based on a fluorescence resonance energy transfer (FRET) assay 107. GSK4112 has been used as an *in vitro* probe of REV-ERBα functions. Note that GSK4112 is not suitable to probe the functions of REV-ERBα* in vivo* due to a low system exposure (poor pharmacokinetic property). Activation of REV-ERBα by GSK4112 inhibits NF-κB signaling and NLRP3 inflammasome activity thus prevents production of cytokines and chemokine, pointing to an anti-inflammatory role of REV-ERBα 25,35,108,109. GSK4112 decreases the viability of 3T3-L1 preadipocytes and reduces the expressions of *cyclin D* (a proliferation-related gene) and β-catenin, revealing a role of REV-ERBα in cell proliferation and apoptosis 87. Also, GSK4112 reduces glycolysis in human gastric carcinoma cells by inhibiting expressions of the genes encoding rate-limiting enzymes 87.

### SR8278

SR8278 is the first synthetic antagonist of REV-ERBs, identified based on Gal4 co-transfection and luciferase reporter assays 110. SR8278 dose-dependently inhibits the transcriptional repressor activity of REV-ERBs 110. However, it has a poor pharmacokinetic property with a very short elimination half-life of around 0.17 h 110,111. SR8278 has been widely used *in vitro* to probe REV-ERBs functions. SR8278 induces expressions of myogenesis genes (*Myod*, *Myog*, and *Mhc3*) in both proliferating and differentiating myoblasts, indicating a regulatory role of REV-ERBs in myogenesis 23. Antagonism of REV-ERBs by SR8278 increases the intracellular level of lactate (reduces glycolysis) in both SGC-7901 and BGC-823 cells 87. There are also several attempts with SR8278 for *in vivo* studies. SR8278 treatment decreases the levels of plasma and liver homocysteine in mice, indicating alleviation of hyperhomocysteinemia by REV-ERBα antagonism 80. SR8278 increases lean mass and improves muscle function in dystrophic mice through activation of Wnt signaling 89.

### SR9009 and SR9011

SR9009 and SR9011 are two potent REV-ERBs agonists designed based on the chemical structure of GSK4112 7,112,113. These two compounds are about threefold more potent and efficacious than GSK4112, and they show better pharmacokinetic properties (may be suitable for* in vivo* studies). In addition, their exclusive actions on REV-ERBs (no effects on other 46 nuclear receptors) have been confirmed by Gal4-chimeric assays. Accordingly, SR9009 and SR9011 have been widely used to test the effects of REV-ERBs on circadian behaviors and diseases both* in vitro* and *in vivo*.

The REV-ERB-specific actions of SR9009 and SR9011 are also supported by loss-of-function studies with REV-ERBα-deficient mice 25,36. SR9009 alleviates DSS-induced colitis and myocardial ischemia-reperfusion in wild-type mice, but fails to do so in REV-ERBα-deficient mice, indicating that the effects of SR9009 are REV-ERBα-dependent 25,36. However, two groups of investigators report potential off-target effects of SR9009 and SR9011. These two agonists show certain LXR activity in the study of Trump et al 114. SR9009 and SR9011 displace a radioligand from the LXRα binding site, and SR9011 increases ABCA1 (a LXR target gene) mRNA in THP-1 cells. In the study of Dierickx et al, SR9009 shows REV-ERBs-independent effects on proliferation, metabolism, and gene transcription in REV-ERBs-deficient mESCs and hepatocytes, although the exact mechanisms for the REV-ERBs-independent effects of SR9009 remain unknown 115.

### GSK2945

GSK2945 was also designed based on GSK4112 scaffold, but shows a superior pharmacokinetic profile with a longer half-life of 2.0 h and an oral bioavailability of 23% 114. This compound dose-dependently represses BMAL1 promoter-driven luciferase reporter activity in U2OS cells, suggesting an agonistic effect on REV-ERBs 114. However, Zhang et al report that GSK2945 dose-dependently antagonizes the repressor activity of REV-ERBα in a Gal4-chimeric assay 76. GSK2945 also represses the Bmal1 reporter activity and blocks the agonistic activity of GSK4112 76. Additionally, the authors demonstrate that GSK2945 increases the mRNA expressions of BMAL1 and PEPCK (i.e., two known target genes of REV-ERBs) in HepG2 cells and hepatocytes as well as in mice 76. Whether GSK2945 is an agonist or antagonist is not conclusive so far. There is a possibility that the action of GSK2945 may be cell/tissue specific as the activities of REV-ERBs can be affected by the cellular microenvironments such as the redox state, small-molecule gasses and the types of cofactors 104,114,116,117. Modifications of ligand-bound REV-ERBs by redox conditions and gasses may result in ligand switching 110.

### ARN5187

ARN5187 directly interacts with the LBD of REV-ERBβ, and acts as an antagonist 91. ARN5187 induces the activity of a RORE-driven luciferase reporter in a concentration-dependent manner, and this effect is lost when RORE is mutated. Additionally, ARN5187 is a dual inhibitor of REV-ERB and autophagy. Application of this dual inhibitor may be an effective strategy for eliciting cytotoxicity in cancer cells.

### Chelidamic acid and bilirubin

Hering et al identified chelidamic acid as a REV-ERBα agonist using a cell-based two-hybrid assay system 118. Chelidamic acid binds specifically to the LBD site of REV-ERBα, leading to enhanced binding of REV-ERBα to the co-repressor NcoR1. Wang et al identified bilirubin, a catabolic product of heme, as an antagonist of REV-ERBα based on Gal4 co-transfection and Bmal1 luciferase reporter assays 119. Despite structurally related, bilirubin and heme display opposite effects on REV-ERBs (i.e., antagonist vs. agonist). Similar findings are also noted for other structurally related compounds (e.g., SR8278 vs. GSK4112; cobalt protoporphyrin IX vs. heme) 110.

### GSK1362

Pariollaud et al developed a novel selective oxazole-based inverse agonist for REV-ERBs, named GSK1362 40. GSK1362 inhibits interactions of REV-ERBα with NCoR1 and SMRT2 peptides according to FRET assays. It also dose-dependently increases Bmal1 promoter-driven luciferase reporter activity in HEK293 cells. Furthermore, the authors established a model for binding of GSK1362 to REV-ERBα with a cellular thermal shift assay, and demonstrated that the O-methyl ethanolamine side chain of the oxazole (forming a key hydrogen bond with Lys473) is crucial for the compound's activity. Of note, GSK1362 does not induce expressions of Abca1 and Abcg1 (two known LXR target genes), suggesting no effects on LXR receptor. Surprisingly, GSK1362 represses LPS-induced *Il-6* in bone marrow-derived macrophages as an REV-ERB agonist does, raising a possibility of an off-target effect.

### SR12418

Amir et al synthesized a REV-ERB-specific synthetic ligand (named SR12418) by modifying the chemical structure of SR9009 33. SR12418 binds to REV-ERBs according to the time-resolved fluorescence resonance energy transfer assay, and shows an exclusive action based on Gal4-chimeric assays. It potently suppresses Bmal1-luciferase reporter activity with an IC_50_ less than one tenth of that of SR9009 (68 nM for SR12418 and 710 nM for SR9009). SR12418 is more effective than SR9009 in inhibiting REV-ERB target genes such as *IL-17*. Moreover, SR12418 displays a better pharmacokinetic property than SR9009. It has been used as an* in vivo* probe to examine the pharmacological effects of REV-ERBs on experimental autoimmune encephalomyelitis and colitis 33.

### Berberine and puerarin

Berberine (initially isolated from *Rhizoma Coptidis*) is reported to be an agonist of REV-ERBα based on three lines of evidence 32. First, berberine inhibits Bmal1-luciferase and Nlrp3-luciferase reporter activities. Second, berberine enhances the REV-ERBα repressor activity in a Gal4 co-transfection assay. Third, treatment of bone marrow-derived macrophages with berberine results in decreased expressions of REV-ERBα target genes. Puerarin is isolated from *Puerariae Radix*, a traditional Chinese medicine widely used to treat fever, emesis, diarrhea, cardiac dysfunctions, and liver injury 120. Chen et al found that puerarin acts as an antagonist of REV-ERBα based on luciferase reporter, Gal4 co-transfection and target gene expression assays 121. Berberine and puerarin differ greatly from other synthetic ligands in chemical structure, indicating discovery of novel chemical scaffolds for REV-ERBα ligands. However, the selectivity of berberine and puerarin toward REV-ERBs has not been validated.

### Other ligands

GSK0999, GSK5072 and GSK2667 were identified together with GSK2945 in the same study 114. These three compounds show similar pharmacokinetic profiles to that of SR9009. Additionally, they have no effects on LXRα. ENA_T5382514, ENA_T5445822 and ENA_T5603164 were identified as REV-ERB ligands in a large-scale screening with 29568 diverse compounds from the Enamine compound library 113. ENA_T5382514 and ENA_T5445822 are agonists, whereas ENA_T5603164 is an antagonist. However, all these three compounds are concerned with off-target effects (e.g., effects on xenobiotic nuclear receptors such as CAR and PXR).

## Promises and challenges

Extensive studies uncover a rather broad role of REV-ERBα in pathological conditions including local inflammatory diseases, metabolic disorders, heart failure and cancers. REV-ERBα ligands have been shown to ameliorate the pathologic conditions in animals (preclinical studies), defining pharmacological activities of these ligands. One prominent advantage of targeting REV-ERBα refers to its pleiotropic effects on multiple cellular and molecular pathways 122. For instance, treatment of diet-induced obese mice with a REV-ERB agonist decreases obesity by reducing fat mass, increasing energy expenditure, and improving dyslipidaemia and hyperglycaemia 7. Despite well-established pharmacological effects of REV-ERBα ligands in animals, no progress has been made regarding their translation to clinical trials. This implies certain challenges associated with drug development of REV-ERBα ligands. Such challenges may include drug safety problem (or adverse effects), suboptimal pharmacokinetics, and potential gap of circadian mechanisms between humans and rodents.

The broad role of REV-ERBα in pathophysiology is a double-edged sword. The broad actions may ensure effectiveness of drugs in treatment of diseases involving multiple cellular and molecular pathways as noted above. However, in terms of drug development, broad actions also mean possible unwanted effects (adverse effects or toxicity). Severe toxicity is one of main cases of drug attrition 123. This is particularly the case for REV-ERBα targeting because activation of REV-ERBα is therapeutically beneficial for certain pathologic conditions (e.g., obesity and inflammations), but is detrimental under other circumstances such as Alzheimer's disease and hyperhomocysteinamia 80,124. Therefore, adverse effects might be a limiting factor to dug development of REV-ERBα ligands.

Suboptimal pharmacokinetic property with poor bioavailability is perhaps another limiting factor for drug development of synthetic REV-ERBα ligands. Current synthetic ligands are cleared rapidly in the body with short half-lives of < 3 h. Because of this, ligands are repeatedly injected daily for more than one week (an unsatisfactory dosing regimen for humans) in efficacy studies with animals. There is a high possibility that these synthetic ligands are rapidly cleared in humans (and even more rapid compared with rodents) and their effectiveness against diseases is impossible to maintain.

Due to an inversed activity-rest cycle between humans and mice (a nocturnal species), serious concerns are raised regarding whether the defined roles of the circadian clock component REV-ERBα in various diseases (and disease-regulatory mechanisms) with mice can be translated to humans. This means that REV-ERBα ligands may be not efficacious at all in humans although they are in animals. Elucidating circadian mechanisms in diseases in humans remains a major task in the field of chronobiology due to the lack of appropriate approaches for extrapolating animal circadian data to humans. It is postulated that advanced models such as humanized animals and primates may address the current gap in circadian studies between humans and mice.

Another issue worthy of attention is that several REV-ERB ligands such as SR9009 have been shown to be “impure”, displaying REV-ERB-independent biological effects (off-target effects). There is a need to determine the selectivity of other REV-ERB ligands (particularly, potential REV-ERB-targeting drugs) and to understand the off-target effects.

## Conclusion

Extensive studies uncover a rather broad role of REV-ERBα in pathological conditions including local inflammatory diseases, metabolic disorders, heart failure and cancers. An array of REV-ERBα ligands have been shown to target REV-ERBα to elicit pharmacological effects. Despite well-established pharmacological effects of REV-ERBα ligands in animals (preclinical studies), no progress has been made regarding their translation to clinical trials. The challenges associated with drug development of REV-ERBα ligands may include safety problem (or adverse effects), suboptimal pharmacokinetics, and potential gap of circadian mechanisms between humans and rodents. For successful drug development, it is suggested that REV-ERBα should be targeted to treat local diseases and a targeting drug should be locally distributed, avoiding the adverse effects on other tissues.

## Figures and Tables

**Table 1:** Target genes of REV-ERBα

Target genes	Type	Species	Refs
*NPAS2*	RCGs	Human	125
*CLOCK*	RCGs	Human	126
*E4bp4/Shp*	RCGs	Mouse	74
*IL-6*	RCGs	Mouse	48
*IL-10*	RCGs	Human	127
*IL-17a*	RCGs	Mouse	33,39
*Nlrp3/p65*	RCGs	Mouse	25,27
*IL-1β*	RCGs	Mouse	27
*Ccl2*	RCGs	Mouse	47
*TLR-4*	RCGs	Human	45
*Mmp9/Cx3cr1*	RCGs	Mouse	16
*PAI-1*	RCGs	Human	128
*Pck1*	RCGs	Mouse	58
*ApoC-III*	RCGs	Human	69
*Elovl3*	RCGs	Mouse	72
*LRH-1*	RCGs	Mouse/Human	76
*Fabp7*	RCGs	Mouse	129
*βKlotho*	RCGs	Mouse	130
*Cyp2b10*	RCGs	Mouse	101
*Ces2*	RCGs	Mouse	98
*Cyp4a*	RCGs	Mouse	100
*Ugt2b*	RCGs	Mouse	99
*Cyclin A*	RCGs	Mouse/Human	88
*PFKFB3/G6PD*	RCGs	Human	89
*PGC1α*	RCGs	Human	131
*Bhmt/Cbs/Cth*	RCGs	Mouse	80
*Ucp1*	RCGs	Mouse	132
*Fmo5*	DCGs	Mouse	17

**Table 2:** Phenotypes of REV-ERBα ablation and the underlying mechanisms

Tissues	Phenotypes	Mechanisms	Refs
Liver	Fulminant hepatitis, IL-1β and IL-18 secretion	NLRP3 inflammasome, Transcription of* Nlrp3* and *IL-1β*	27
Liver	Diabetes	Transcription of *PCK* and *G6Pase*	6, 57-61
Liver	Hyperlipidemia	Transcription of *ApoC-III* and *Elovl3*	6,69,70,72
Liver	Hypercholesterolemia	Expression of HMGCR and CYP7A1	71,74-76
Liver	Hyperhomocysteinemia	Transcription of *Bhmt*, *Cbs*, *Cth* and *C/EBPα*	80
Lung	Pulmonary inflammation	Expression of chemokines and inflammatory cytokines	40
Lung	Pulmonary fibrosis	TBPL1-integrinβ1 pathway	56
Colon	Ulcerative colitis	NF-κB signaling pathway, NLRP3 inflammasome, Transcription of* Nlrp3*	25
Heart	Ischaemia-reperfusion injury, Immunocyte recruitment	NLRP3 inflammasome	36
Heart	Perioperative myocardial injury	Expression of CDKN1a/p21.	30
Bone	Osteoporosis, Osteoclastogenesis	Expression of FABP4	84
Bone	Osteogenesis	Expression of bone sialoprotein	85
Pancreas	Glucose-induced insulin secretion in β-cells, Glucagon secretion in α-cells	Exocytotic process, AMPK/Nampt/Sirt1 pathway	62-64
